# Automatic facial emotion recognition at the COVID-19 pandemic time

**DOI:** 10.1007/s11042-022-14050-0

**Published:** 2022-10-22

**Authors:** Giovanna Castellano, Berardina De Carolis, Nicola Macchiarulo

**Affiliations:** 1 Department of Computer Science, University of Bari, Bari, Italy; 2 Exprivia S.p.A., Molfetta, Italy

**Keywords:** Facial expressions recognition, Masked face, Emotions

## Abstract

People use various nonverbal communicative channels to convey emotions, among which facial expressions are considered the most important ones. Thus, automatic Facial Expression Recognition (FER) is a fundamental task to increase the perceptive skills of computers, especially in human-computer interaction. Like humans, state-of-art FER systems are able to recognize emotions from the entire face of a person. However, the COVID-19 pandemic has imposed a massive use of face masks that help in preventing infection but may hamper social communication and make the recognition of facial expressions a very challenging task due to facial occlusion. In this paper we propose a FER system capable to recognize emotions from masked faces. The system checks for the presence of a mask on the face image and, in case of mask detection, it extracts the eyes region and recognizes the emotion only considering that portion of the face. The effectiveness of the developed FER system was tested in recognizing emotions and their valence only from the eyes region and comparing the results when considering the entire face. As it was expected, emotions that are related mainly to the mouth region (e.g., disgust) are barely recognized, while positive emotions are better identified by considering only the eyes region. Moreover, we compared the results of our FER system to the human annotation of emotions on masked faces. We found out that the FER system outperforms the human annotation, thus showing that the model is able to learn proper features for each emotion leveraging only the eyes region.

## Introduction

Humans employ different cues to express their emotions, such as facial expressions, voice intonation, gestures, and postures. According to [25], 55% of the messages are conveyed through facial expression, 7% through words, and 38% through paralanguage like speech intonation. Then the face is the main channel people use to decipher the feelings of others since it is the prime communicator of emotion [30]. What makes the communication of emotions through the face interesting is that it appears as if some of these expressions of emotion (e.g., anger, disgust, fear, happiness, sadness, surprise) are universal [9]. The interest in emotion recognition is increasing especially in human-computer interaction research in order to enrich the capability of a machine to perceive the feelings of its users. Most existing research works focus on Automatic Facial Expression Recognition (FER) based on Ekman’s theories, which suggests that there are six basic emotions universally recognized in all cultures: happiness, surprise, anger, sadness, fear, and disgust [9]. FER is of fundamental importance in various applications, such as intelligent tutoring systems [41], interactive game design [21], affective robots [1], driver fatigue monitoring [2], personalized services [38], and many others.

The problem of recognizing emotions during the COVID pandemic has recently gained a lot of interest because, from a psychological perspective, the pandemic introduces uncertainty and anxiety in people. These aspects may increase during social isolation and can result in depressive symptoms and anxiety. As found out by the study in [26], the confinement situation experienced during COVID-19 can affect emotion recognition because, due to isolation, interactions and social contacts are drastically reduced, so there are differences in facial emotion recognition in people confined for COVID-19 and people unconfined. Therefore, recognizing emotions during a pandemic is of fundamental importance for health care providers to detect these emotional outcomes and identify as soon as possible the appearance of psychological consequences in people affected by the COVID-19 disease.

A major concern in recognizing facial emotions during a pandemic is the presence of the mask that occludes a large part of the face, thus representing a big challenge for a FER system. Actually, FER systems developed so far have shown high performances in constrained environments. On the contrary, emotion recognition in real-life conditions is still a challenging task due to several factors such as pose variations, different age, gender, or culture of the subject, and especially face occlusion. With the advent of the COVID-19 pandemic, occlusion has become a constant condition in face-to-face communication since face masks are used in both indoor and outdoor environments as personal protective equipment and their use is often declared compulsory by authorities to slow down infections.

Recognizing facial expressions in presence of face masks is difficult even for humans because, unless the masks are transparent [23], the lower part of the face is occluded. In [6] the authors highlighted how the face masks affect the reading of the emotions. Covering about 60-70% of the face, humans make mistakes in understanding disgust (recognized as anger), happiness, and sadness (recognized as neutral). Similar results were found in [12]. The mouth region is a very informative area for recognizing emotions, in fact, surprise and fear require the analysis of the mouth to be distinguished by humans, as well as disgust and sadness. Another social study on the impact of face masks on emotion recognition and social judgments (perceived trustworthiness, likability, and closeness) was carried out in [14]. Such a study confirms that humans are less accurate in emotion recognition in the presence of masked faces. Nevertheless, the region of the eyes is very crucial in expressing and recognizing emotions and it may be informative enough to carry out a FER analysis.

Starting from this insight, in our previous work, [7] we developed a FER system to automatically recognize facial emotions even in presence of masks. Such a system is based on the combination of two deep learning models for emotion recognition: one model is trained on eyes regions and is activated when a face mask is detected, and the other model is trained on unmasked faces and is activated when no mask is detected. The purpose of our preliminary work [7] was to understand the extent to which a FER system can be effective in recognizing emotions from masked faces and to identify which emotions are confused with others when considering only the eye region.

In this paper, we extend our preliminary work [7] to investigate whether the use of the attention mechanism inside deep learning models can improve the results in terms of recognition accuracy. Specifically, the novel contributions of this paper with respect to [7] are the following:

1. we develop a FER system based on deep learning models equipped with attention;
2. we perform an experiment on the recognition of the emotion’s valence;
3. we make a comparison between the proposed FER system and human annotations.

The empirical validation carried out in the present work was done to answer the following research questions:

- Q1: Can a convolutional neural network with an attention mechanism improve the FER accuracy in presence of a face mask?
- Q2: Is the accuracy of our FER system comparable to human accuracy in the classification of emotions from masked faces?

As concerns Q1, we found that embedding attention in a convolutional neural network leads to an improvement in FER accuracy but only on unmasked faces. As the emotion valence is concerned, the approach achieves a better performance in recognizing positive emotions than negative ones. As concerns Q2, we found an agreement between the FER system and the human annotation of 51% for emotion recognition. Except for sadness, the FER system outperforms the human annotation in classifying all other emotions, thus showing that, similarly to children growing up in the era of the COVID-19 pandemic [32], the model is able to learn proper features for each emotion leveraging only the eyes region. In general, the obtained results show that our FER system has a good performance in recognizing positive emotions and highlights a great improvement in recognizing negative emotions even in presence of a facial mask.

The paper is organized as follows. Section 2 describes research works addressing the same problem. Section 3 describes the pipeline designed and developed for our FER system. In Section 4 we show the experimental results. Finally, Section 5 draws the conclusions and future work.

## Related work

Facial Expression Recognition is the task of predicting a specific facial expression given a facial image. FER has demonstrated remarkable progress due to the advancement of deep learning. One of the problems in emotion recognition from facial expressions regards occlusion, which recently has become a constant condition due to the COVID pandemic that requires wearing facial masks. In this case, recognition of emotions from facial expressions has become more difficult even for humans because the lower part of the face is occluded. The problem of facial occlusion was already addressed in the literature [4], and it was found that even the use of scarves [20] and sunglasses [31] makes emotion recognition more difficult.

Several social studies have been presented on the impact of face masks on the human ability to recognize facial emotions [6, 14, 27, 29]. These studies mainly show that many people tend to rely on the eyes to discern a face’s emotional expression rather than the mouth or nose. However, there are very few attempts to analyze the capability of automatic FER systems in recognizing emotions in presence of facial masks.

A number of studies have been conducted on FER in presence of partial occlusion [42], but a few works focus on the specific case of occlusion due to a mask because of its highly challenging nature. Indeed, unlike other partial occlusion FER problems, wearing a mask covers half of a person’s face, especially the mouth, which is highly informative to distinguish between the emotions of sadness and disgust, or fear and surprise [36].

Besides our previous work [7], to the best of our knowledge, only a few works address the automatic FER in presence of masks. In [35] a FER system is built using Convolution Neural Networks (CNN) that is trained on eyes and forehead segments. In [39] the authors propose a two-stage attention model to improve the accuracy of face-mask-aware FER: in the first stage, they train a masked/unmasked binary deep classifier, which generates attention heatmaps to roughly distinguish the masked facial parts from the unobstructed region; in the second stage the FER classifier is created to pay more attention to the region that is essential to the facial expression classification, and both occluded and non-occluded regions are taken into consideration but reweighed.

One main problem in developing FER models is that no existing datasets are specified for masked FER technology, and none of them considers facial orientation. To overcome this problem, some works consider masked versions of FER datasets to create their models, similarly to our previous work [7]. In [40] the authors propose a method that can add face masks to existing FER datasets automatically using differently shaped masks according to facial orientations. The FER models based on VGG19 and MobileNet are trained on public and private FER datasets added with a mask. Moreover, in [40] the authors collected real-world masked faces from the Internet using emotional keywords and constructed a masked FER test dataset for a fair performance evaluation using various masks of different colors and shapes while also taking facial orientations into account. Similarly to [40], in [7] and in the present work, we apply crowd-sourcing to collect images of masked faces annotated by humans and create a dataset for FER assessment. However, in [40] the authors consider only the classification of the emotion valence, i.e. positive, neutral, and negative, which is a quite simpler task. In our work, we collect and annotate images with expressions in the basic emotions space made by seven labels (i.e. angry, disgust, fear, happy neutral, sad, and surprise). In [37] a deep CNN is trained on the masked version of the JAFFE dataset, showing that the accuracy of recognizing emotions from masked faces is lower than the accuracy of recognizing emotions from unmasked faces. Likewise, in [3] the authors added a mask on the faces in the AffectNet dataset and trained a CNN in different settings in order to estimate valence and arousal. Greco et al. [13] performed an experimental analysis to evaluate the performance gap of several face analysis tasks. In particular, for the emotion recognition in presence of a mask the authors used for their experiments the RAF-DB-M dataset, that is the dataset RAF-DB with the digital addition of the facial mask. The accuracy of the approach is 46.5% on the validation set with mask, a drastic drop if compared to the accuracy obtained with the unmasked faces (85.7%).

Finally, similarly to our approach, the method described in [10] embeds attention mechanisms into a ResNet50-based architecture to recognize emotions even in presence of facial masks. The experiments were conducted both on unmasked and masked faces, showing some degree of robustness in emotion recognition when the face is occluded.

As a final remark, it should be noted that all the above works consider the whole face for emotion recognition, even in presence of a mask. This may cause side effects in the behavior of the FER models because a face mask can be interpreted as noise that hampers the task of emotion recognition. On the contrary, our method considers only the region of the eyes when a mask is detected, so as to better focus on proper visual features for the FER task.

## The FER system

Generally, a FER system is composed of three main steps: preprocessing, feature extraction, and classification task that maps the extracted features to a label space comprising the basic emotions plus the neutral one.

**Fig. 1:**
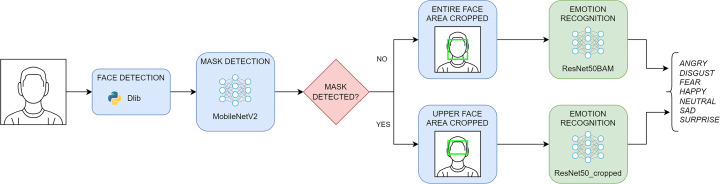
The designed pipeline for facial emotion recognition performed both with and without a mask

To accomplish FER in presence of a face mask, we modify the basic pipeline by adding a mask detection phase, as depicted in Fig. 1. After detecting the face of a person in the image, we determine the presence of a mask using a CNN trained for this purpose. If a mask is detected, only the part of the face around the eyes is considered as a Region Of Interest (ROI). In case the mask is not present, the entire detected face is considered as a ROI. Then, the emotion recognition module analyzes the ROI and applies another CNN to classify the emotion into one of the following ones: happiness, surprise, anger, sadness, fear, disgust, and neutral.

The main phases of the proposed pipeline are detailed in the following sections.

### Face detection

A face detection step is first performed using the frontal face detector of the Python library *Dlib* 1 [18]. The detected region is cropped with size 224x224 to be compatible with the input size accepted from the convolutional neural network that performs mask detection (Section 3.2).

### Mask detection

The next step consists in checking the presence of a mask on the face detected in the previous step. For this purpose, we used a CNN based on MobileNetV2 [34] pretrained on ImageNet [33]. The top layers were replaced with the ones useful for mask detection. Specifically, an average pooling operation and two fully connected layers were added after the convolutional blocks. The fine-tuning of the CNN was performed using a dataset for mask detection available on Github 2. This dataset consists of 1376 pictures of people’s faces, with 686 images without mask and 690 with mask. This set of masked face images was produced artificially. Indeed, the authors of the dataset added a mask to the people’s faces by detecting their facial landmarks and positioning the image of the mask in the correct location. The dataset was then split into a training set (80%), a validation set (10%), and a test set (10%). The Adam optimizer [19] was used to train the neural network with a learning rate of 0.0001, a batch size of 32, and a number of epochs of 100. We also adopted a learning rate reduction strategy by decreasing the learning rate by a factor of 10 every 5 epochs without accuracy improvement. These values for hyperparameters were obtained empirically by carrying out several training runs with different values.

The accuracy achieved on the test set by the model was 96%. In particular, the model made just a few errors (6/84) in detecting the presence of the mask, thus it can be considered quite reliable for this task.

### Emotion recognition

For the emotion recognition task, two different models were developed and alternatively activated on the basis of the result of the mask detector module.

**Fig. 2:**
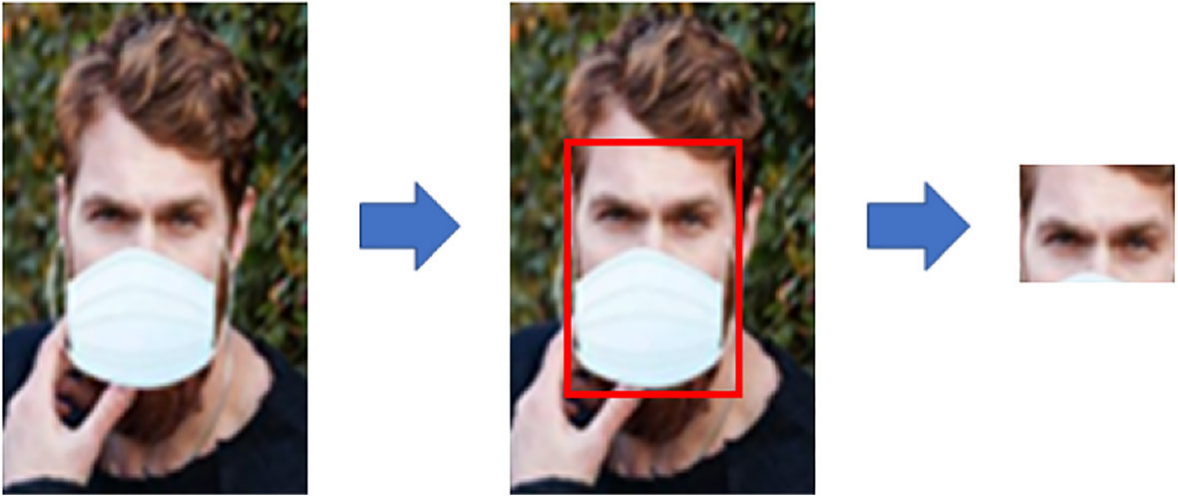
Crop of the region of interest when a facial mask is detected

The first model performs the emotion recognition task on the entire face in case the mask is not detected. The second model is activated in case of mask detection and considers only the ROI that includes the eyes (Fig. 2) for the emotion recognition. In both cases, we resized the images to 224x224, to be compatible with our models.

#### The Datasets

The two models were trained respectively on two datasets extracted from the FER2013 dataset that was created in 2013 by the organizers of the “Challenges in Representation” competition [11]. The FER2013 dataset contains 35887 grayscale images of people’s faces resized to 48x48 pixels. Each image is annotated with one of the following emotions: *Angry, Disgust, Fear, Happy, Neutral, Sadness, and Surprise*. The dataset is already divided into a training (28709 images), a validation (3589 images), and a test set (3589 images). We chose the FER2013 dataset because it is considered a benchmark in facial expression recognition and it contains bot posed and unposed head photoshoot.

Starting from the FER2013 dataset, we created a new one, called *FER2013_filtered*, to be used for learning the FER model for unmasked faces. Specifically, we considered only the images of the FER dataset for which it was possible to detect the face landmarks. In this way, the number of images per set was reduced to 26427 for training, 3335 for validation, and 3333 for test.

**Fig. 3:**
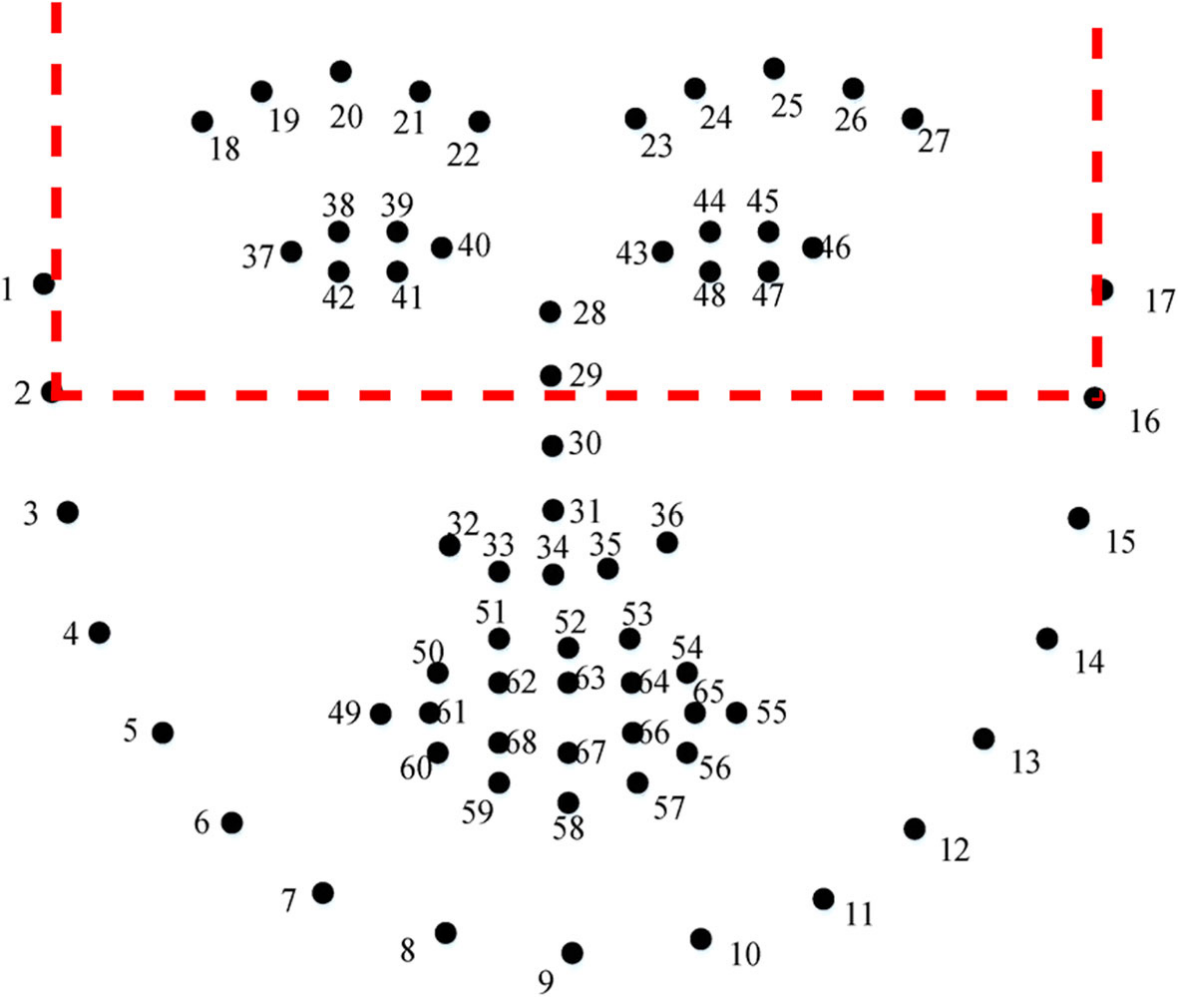
The region of interest of the upper face area based on the 68 facial landmarks

We also created a second dataset, named *FER2013_cropped*, to be used for creating the FER model for masked faces. This dataset was created by cropping each image of the *FER2013_filtered* dataset so as to include only the eyes area which is the visible area when wearing a mask. More precisely, the ROI was determined by considering the 68 facial landmarks *FL*_*i*_,*i* = 1,...,68 computed by the face detector (Fig. 3) and selecting a rectangle that originates in the point (*x*,*y*) defined as:

$$x = FL_{1}.x, y = max(FL_{20}.y, FL_{25}.y)$$

and has width *w* and height *h* defined as:

$$w = FL_{17}.x, h = min(FL_{2}.y, FL_{16}.y)$$

**Fig. 4:**
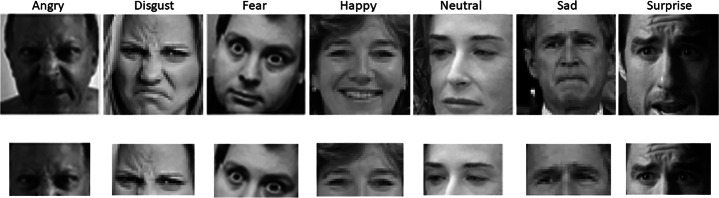
The first row contains the images extracted from the *FER2013_filtered* dataset. The second row contains the cropped version of the same images in the *FER2013_cropped* dataset

The emotion label associated with the original image was also associated with the cropped version. Figure 4 shows examples of facial images from both the *FER2013_filtered* and *FER2013_cropped* datasets.

#### The models

Our FER system, as shown in Fig. 1, can follow one of the two different paths after the mask detection phase in order to use alternatively two classification models, one for recognizing emotions in absence of mask and one for recognizing emotions in presence of mask. We compared the results of two different models for each path: a CNN based on the state-of-the-art ResNet50 architecture [16] pretrained on the VGGFace2 dataset [5], which is a dataset used for face recognition and it contains 3.31 million images, and the same architecture with the addition of attention modules. In this work, we chose the module called Bottleneck Attention Module (BAM) [28], in particular, we placed three BAMs at the end of the first three bottlenecks of the model. For *the FER2013_filtered* dataset, we trained two models named *ResNet50* and *ResNet50BAM*. The same approach was followed for *FER2013_cropped* dataset and two models were trained, named *ResNet50_cropped* and *ResNet50BAM_cropped*. The training phase was performed for 100 epochs with the Adam optimizer, a learning rate of 0.001, and a batch size of 64. Also in this experiment, the learning rate reduction strategy described in Section 3.2 was adopted.

## Experimental results

We performed two experiments. The first experiment aimed to compare the accuracy of the models underlying the FER system, i.e. the models capable to recognize emotion in presence of occlusion due to the mask were compared with the models trained to recognize the emotion from the entire face. The second experiment aimed at comparing the automatic recognition of emotions in masked faces to the one made by human annotators.

### Recognition of emotion in masked faces

In our work, we consider the mask as a simple occlusive element of the lower part of the face. As well known, facial expressions might be affected by the mask, however, we have not considered this aspect in our work, thus we assumed that an emotion is expressed in the same way both with and without a mask.

The following measures were computed in order to compare the FER models trained on the dataset with mask and on the dataset without mask:

- $Precision=\frac {TP}{TP+FP}$
- $Recall=\frac {TP}{TP+FN}$
- $F1=\frac {2*(Recall * Precision)}{(Recall + Precision)}$
- $Accuracy=\frac {TP+TN}{TP+FP+FN+TN}$

**Fig. 5:**
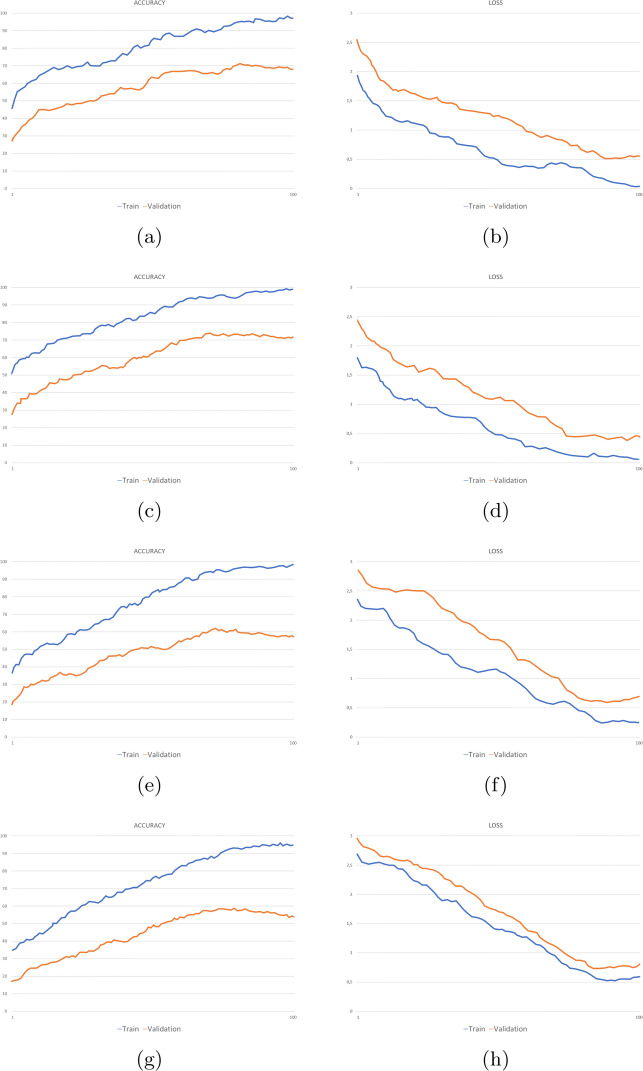
Accuracy and loss values during training of *ResNet50* (a, b), *ResNet50BAM* (c, d), *ResNet50_cropped* (e, f), and *ResNet50BAM_cropped* (g, h)

TP denotes the number of true positives, TN indicates the number of false positives, the number of false positives is represented with FP and the number of false negatives is denoted with FN. These measures were used to calculate the confusion matrix. The comparative results of the models without attention mechanisms are summarized in Table 1. In Table 2 the performances achieved by the models with attention modules are compared. Figure 5 shows the accuracy and loss values measured during the training phase of each experimental setting.

**Table 1:** Accuracy metrics of the models *ResNet50* and *ResNet50_cropped* in the two cases: without mask (*FER2013_filtered*) and with mask (*FER2013_cropped*)

Emotions	Precision		Recall		F1-score		Accuracy	
	*Filt.*	*Crop.*	*Filt.*	*Crop.*	*Filt.*	*Crop.*	*Filt.*	*Crop.*
*Angry*	**0.64**	0.56	**0.67**	0.56	**0.65**	0.56	**0.67**	0.56
*Disgust*	0.70	**0.77**	**0.67**	0.52	**0.69**	0.62	**0.67**	0.52
*Fear*	**0.60**	0.53	**0.54**	0.48	**0.57**	0.50	**0.54**	0.48
*Happy*	**0.91**	0.79	**0.92**	0.82	**0.91**	0.80	**0.92**	0.82
*Neutral*	**0.75**	0.61	**0.74**	0.61	**0.74**	0.61	**0.74**	0.61
*Sad*	**0.58**	0.48	**0.59**	0.51	**0.58**	0.49	**0.59**	0.51
*Surprise*	**0.81**	0.76	**0.84**	0.75	**0.82**	0.76	**0.84**	0.75

The best performance metrics for each emotion are in bold

**Table 2:** Accuracy metrics of the models *ResNet50BAM* and *ResNet50BAM_cropped* in the two cases: without mask (*FER2013_filtered*) and with mask (*FER2013_cropped*)

Emotions	Precision		Recall		F1-score		Accuracy	
	Filt.	Crop.	Filt.	Crop.	Filt.	Crop.	Filt.	Crop.
Angry	**0.67**	0.55	**0.68**	0.53	**0.67**	0.54	**0.67**	0.53
Disgust	**0.77**	0.72	**0.69**	0.54	**0.73**	0.62	**0.69**	0.54
Fear	**0.63**	0.56	**0.54**	0.43	**0.58**	0.49	**0.54**	0.43
Happy	**0.90**	0.75	**0.93**	0.83	**0.91**	0.79	**0.93**	0.83
Neutral	**0.75**	0.59	**0.76**	0.64	**0.76**	0.61	**0.76**	0.64
Sad	**0.59**	0.49	**0.63**	0.48	**0.61**	0.49	**0.63**	0.48
Surprise	**0.83**	0.72	**0.82**	0.74	**0.83**	0.73	**0.83**	0.74

The best performance metrics for each emotion are in bold

It can be noticed that, in both cases, the global accuracies of the models trained on *FER2013_cropped* (63.52% for *ResNet50_cropped* and 62.62% for *ResNet50BAM_cropped*, both computed with weighted accuracy) are lower than the accuracies of the models trained on the *FER 2013_filtered* dataset (73.21% for *ResNet50* and 74.32% for *ResNet50BAM*, both computed with weighted accuracy). Another observation concerns the effectiveness of the attention modules. On the *FER2013_filtered* dataset, that is the dataset containing the images of the entire face, the model without attention mechanisms (*ResNet50*) achieved an accuracy of 73.21%, a lower performance than the model with BAM (*ResNet50BAM*).

On the other hand, opposite results were obtained on the dataset *FER2013_cropped*. In fact, the model without attention (*ResNet50_cropped*) achieved higher accuracy than *ResNet50BAM_cropped*. This may be due to the image cropping, in particular, the crop of the region of the eyes could already be considered as an attempt to focus the attention on the most important region of a masked face. Considering the higher results obtained by the *ResNet50_cropped* model, we will consider it as the model used in our pipeline after a masked face is detected. Therefore, further analysis will concern only this model. If we divide the emotions recognized by their valence (positive or negative), we can notice that the average accuracy of the positive emotions (*Happy* and *Surprise*: 79.8%) is greater than the average accuracy of the negative ones (*Angry, Disgust, Fear* and *Sad*: 51.42%). Therefore, the model *ResNet50_cropped* is able to better recognize the positive emotions in presence of occlusion.

**Fig. 6:**
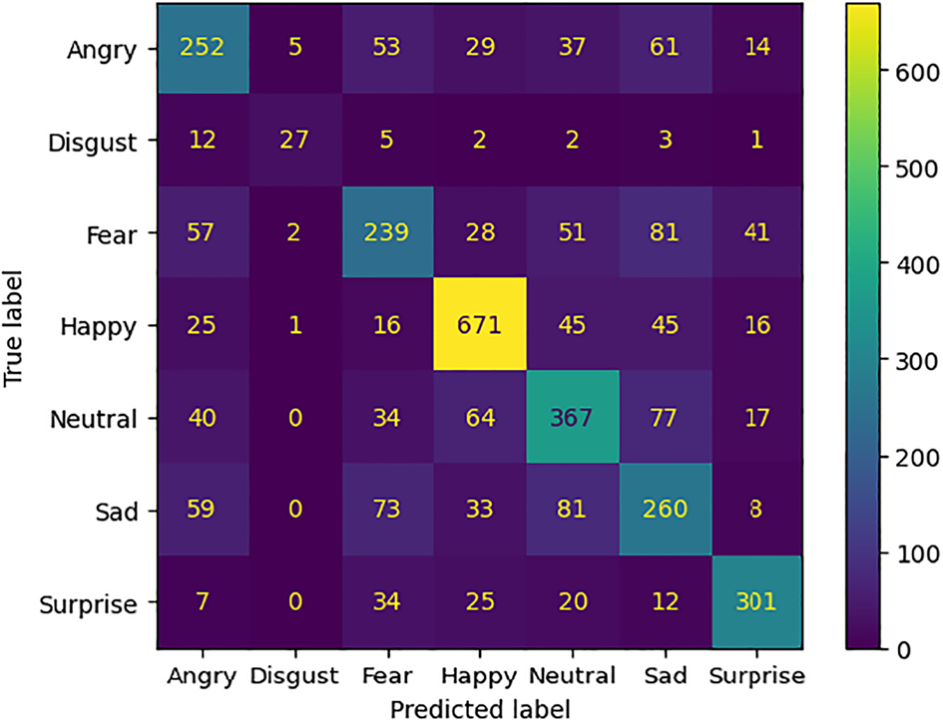
Confusion matrix of the model *ResNet50_cropped* for emotion recognition with the mask (*FER2013_cropped*)

**Fig. 7:**
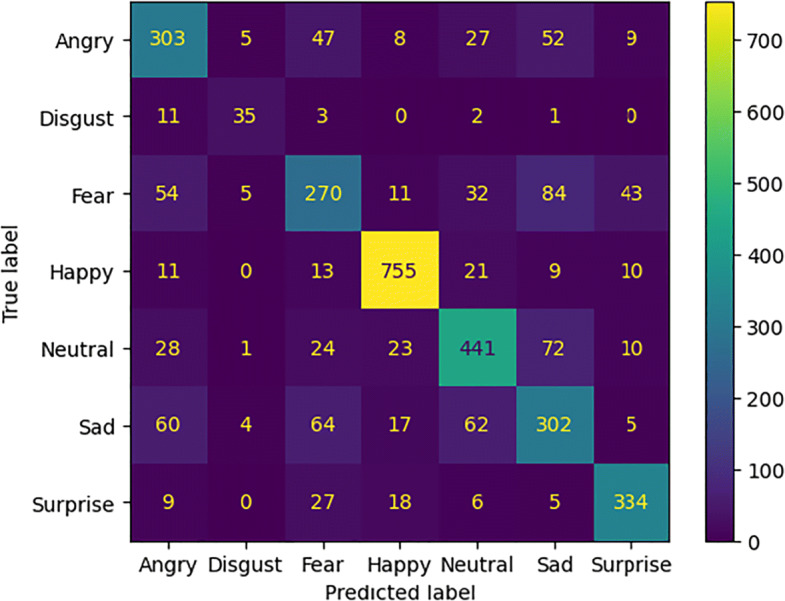
Confusion matrix of the model *ResNet50* for emotion recognition without the mask (*FER2013_filtered*)

**Fig. 8:**
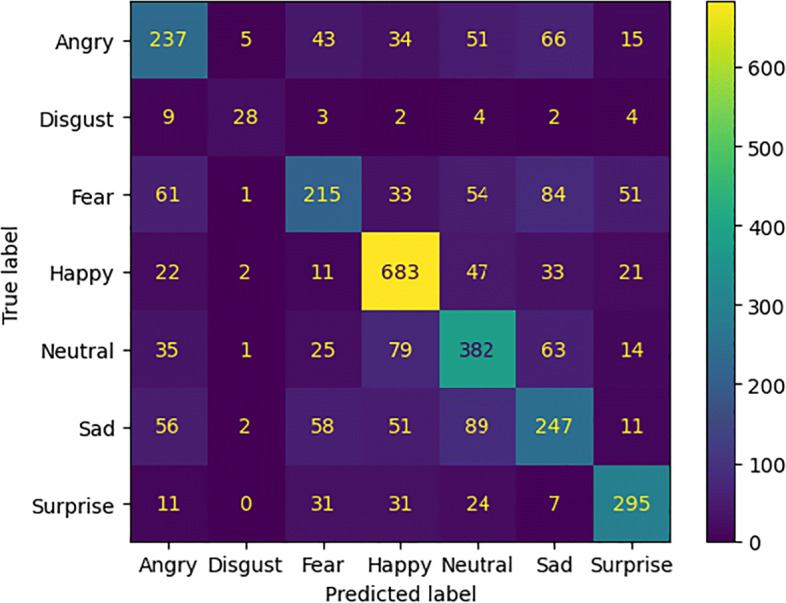
Confusion matrix of the model *ResNet50BAM_cropped* for emotion recognition with the mask (*FER2013_cropped*)

**Fig. 9:**
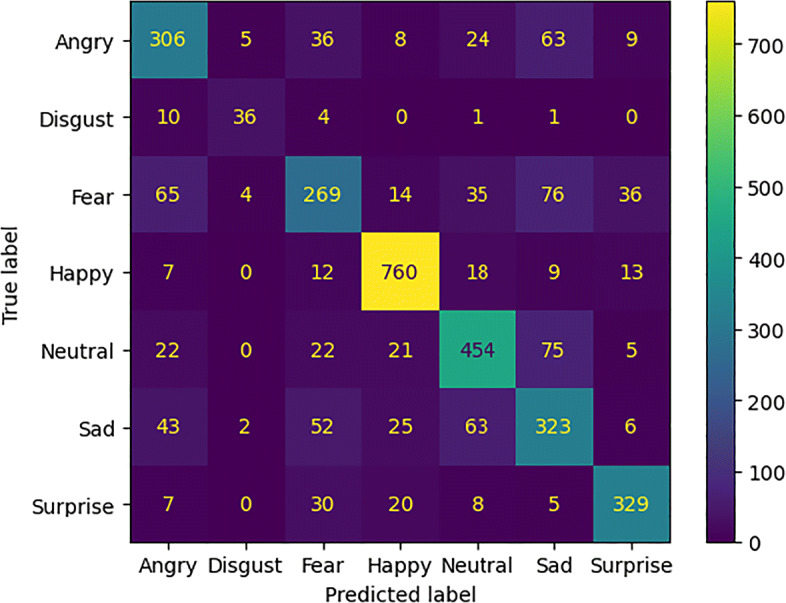
Confusion matrix of the model *ResNet50BAM* for emotion recognition without the mask (*FER2013_filtered*)

Considering the confusion matrices in Figs. 6, 7, 8, and 9 we can notice that both models, just like humans do [6], confuse the emotions with a negative valence with each other. The *Happy* emotion is recognized with the highest accuracy than the others even with the presence of the mask.

**Fig. 10:**
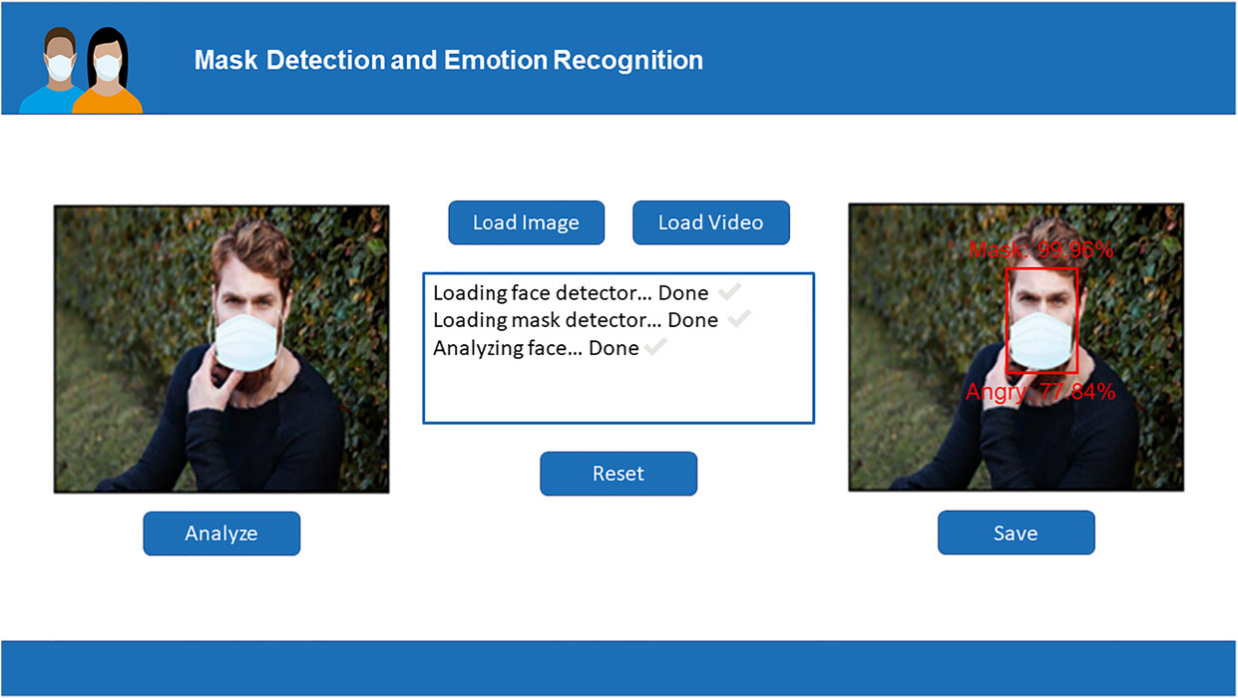
The interface of the desktop application developed and an example of the analysis performed on the loaded image

We deployed our FER pipeline in a real-time desktop application that is able to analyze new images or videos and recognize the emotion expressed by the face. The models *ResNet50BAM* and *ResNet50_cropped* were used to classify the emotion in the absence or presence of the mask, respectively. Figure 10 shows the application interface. On the left side, the original image or video is shown, while, on the right side, the box surrounding the face, the mask detection result, and the emotion recognized are shown. In the example, the presence of the mask is detected and the *Angry* emotion is correctly recognized.

### Recognition of emotion’s valence

Following the findings in Section 4.1, we performed an experiment on the recognition of the emotion’s valence. For this purpose, we trained the same ResNet50 based model without attention described in Section 3.3.2 on the dataset M-LFW-FER [40]. This dataset is based on the dataset LFW [17] and it contains 10462 images labelled with one of the following valence values: negative, neutral, and positive. As explained in Section 3.3.1, we adopted the same preprocessing method to crop the upper part of the faces when mask is detected. Table 3 shows the performance of the model in recognizing emotion’s valence and Table 4 reports its confusion matrix.

**Table 3:** Accuracy metrics of the ResNet50 based model on the validation set of M-LFW-FER

Valence	Precision	Recall	F1-score	Accuracy
*Neutral*	0.69	0.83	0.75	0.83
*Negative*	0.61	0.33	0.42	0.33
*Positive*	0.88	0.82	0.85	0.83

**Table 4:** Confusion matrix of the ResNet50 based model on the validation set of M-LFW-FER

	Neutral	Negative	Positive
*Neutral*	344	14	58
*Negative*	51	31	13
*Positive*	107	6	531

The model shows good performance in recognizing neutral and positive facial expressions from the upper face region. There is a drastic worsening in the recognition of negative expressions. Most of the negative expressions are incorrectly classified as neutral expressions. The behavior of the model is the same as the model *ResNet50_cropped*, thus confirming that negative expressions are also strongly related to the lower part of the face.

We also compared the global accuracy obtained by the model with the one obtained by the baseline described by the authors of the dataset. The baseline model is based on MobileNet and achieved an accuracy of 0.72. With our model, we obtained a higher accuracy (0.78) with an improvement of + 0.06.

### Comparing automatic and human emotion recognition when wearing a mask

To better assess the capability of the automatic classification of emotion on masked faces obtained by our FER system, we made a comparison with the human annotation.

Generally, psychologists have proposed two approaches to study nonverbal behavior (including facial expressions), either judgement-based or sign-based [15]. In judgement-based approaches, humans look at the image of the face and make inferences about the expressed emotion and assign corresponding labels. When classifying facial expressions into a predefined number of emotions, the agreement of the annotators is taken as ground truth, usually by computing the average of the responses. The idea is that the facial expression in the image should be universally understood by a human, or it would be useless and therefore it should be removed by the annotation task. Thus, crowd-sourced annotation, a practice that uses many humans to label a target image, may be a useful way for this task.

Then, as a first step, we randomly sampled 43 images for each class from the test set of the *FER2013_cropped* dataset, thus collecting a total of 301 images. Subsequently, for the annotation phase, we developed a web-based crowd-sourcing application to gather image annotations quickly and economically.

**Fig. 11:**
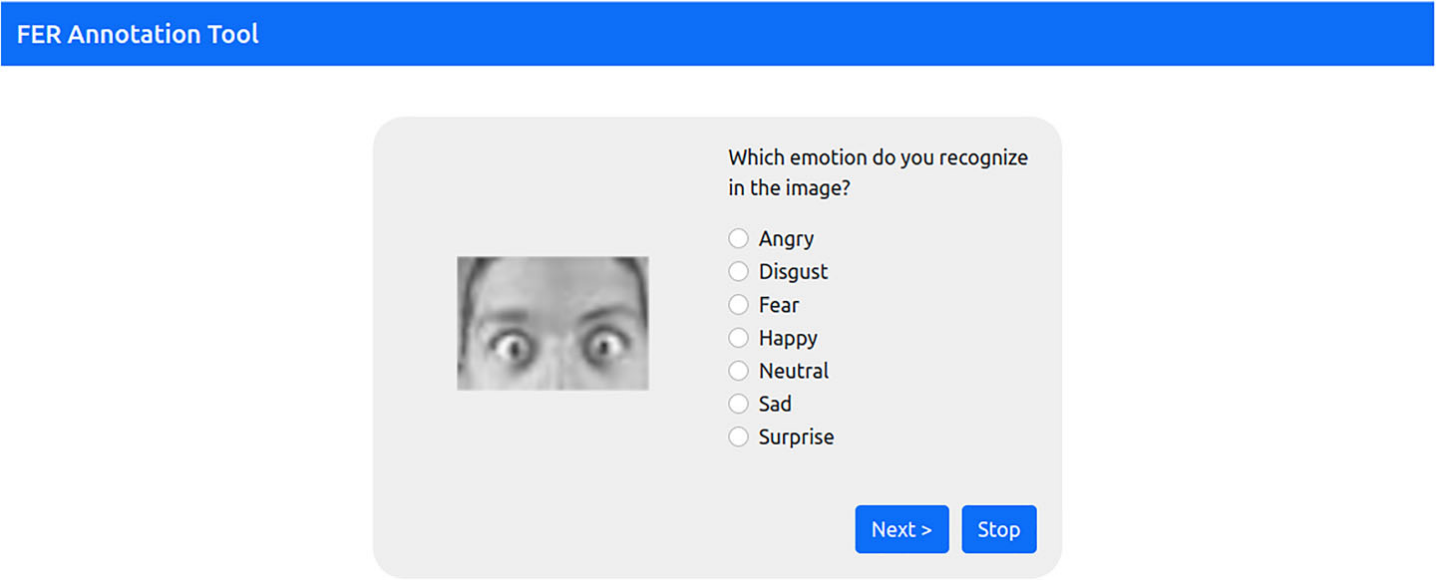
The interface of the web application used for the annotation task

People were invited to use the application through instant messaging or social media. The annotation interface is shown in Fig. 11, the layout is very simple, the image is shown on the left and the annotators can select the perceived emotion on the right.

We made the web application available on the web until each image got 5 annotations. The annotation process was totally anonymous and this phase lasted 34 days. 81 people (35 males and 46 females) participated in this process and, on average, each one annotated 19 images for 1539 annotations in total.

In analyzing the data, first of all, we considered the majority agreement criteria, so only the images that received at least 3/5 votes on the same emotion were selected, and the others were discarded. Only 24 images did not reach the majority quota, therefore 277 images were selected in this step. Then two expert psychologists, expert in communication, assigned to each image the emotion label by supervising the results of the majority vote.

The agreement between human annotation and the system prediction is 51%, which indicates that in almost half of the cases annotators gave a different label compared to the one assigned by the automatic classifier.

Then, we measured the accuracy of the human annotation with respect to the emotion label assigned to the 277 images in the FER 2013 dataset. The accuracy achieved by the human annotation is 49%, and the performance metric for each class is reported in Table 5. In the same table, we also reported the accuracy of the FER system. It can be seen that its global accuracy is higher than the accuracy reached by human annotators. The higher performances of the model are evident for the classes *Disgust* (+ 0.20), *Fear* (+ 0.31), *Happy* (+ 0.20), *Neutral* (+ 0.06), and *Surprise* (+ 0.13). For *Angry*, the accuracy is almost the same. Instead, the human annotation can better recognize the emotion *Sad* (+ 0.07).

**Table 5:** Accuracies of the human annotation and the FER system classification

Emotions	Human Accuracy	FER System Accuracy
*Angry*	**0.57**	0.56
*Disgust*	0.32	**0.52**
*Fear*	0.17	**0.48**
*Happy*	0.62	**0.82**
*Neutral*	0.55	**0.61**
*Sad*	**0.58**	0.51
*Surprise*	0.62	**0.75**
*Global*	0.49	**0.63**

The highest accuracy for each emotion is in bold

To better verify the agreement between the ground truth and the annotations given by the model and the human annotators, we computed the Cohen’s kappa coefficient (k) [8, 24]. The *k* statistic measures the inter-rater reliability for qualitative categorical items and takes into account the possibility of the agreement occurring by chance. We computed this statistic both on the seven basic emotions annotation and on the emotion’s valence annotation. The results obtained are reported in Table 6. It can be noticed that, in general, the agreement computed on the emotion’s valence annotations is higher than the one obtained on the seven basic emotions annotations. The agreement between human annotations and ground truth is moderate. Instead, there is a good agreement between the response of the model and the ground truth. This result confirms the higher accuracy obtained by the model compared to human annotation and shows that it is not due to chance.

**Table 6:** Cohen’s kappa coefficient

Annotators	Annotation	k
*Human - Ground Truth*	Seven basic emotions	0.40
	Emotion’s valence	0.48
*Model - Ground Truth*	Seven basic emotions	0.55
	Emotion’s valence	0.63
*Human - Model*	Seven basic emotions	0.43
	Emotion’s valence	0.45

## Conclusions and future work

Automatic facial expression recognition is a very important feature for systems that try to simulate human interaction. It can be used in various scenarios, such as e-learning, marketing, healthcare, and social robotics. In this period of the Coivd-19 pandemic, recognition of emotions from facial expression is impaired by the use of a mask that covers more than half of a person’s face.

In this study, we investigated how effective is a FER system in recognizing emotions in masked faces by comparing the same approach to the recognition of the same expressions on the entire face and then we compared its performance to humans’ accuracy in recognizing emotions from masked faces.

The proposed pipeline is based on deep learning techniques and the adoption of a more sophisticated model improved the global accuracy in recognizing emotion even from only the upper part of the face. In general, the accuracy measured on the occluded faces is only about 10% lower with respect to the accuracy measured on the entire face. Emotions with positive polarity are better recognized because they are strongly related to the region of the eyes. The improvement of negative emotions recognition is of about 30% respect our initial work proposed. The FER system still confuses some negative emotions, for example, *Angry* is often classified as *Fear* or *Sad* and vice-versa or *Disgust* is misclassified as *Angry*. However, when comparing the accuracy of the FER system with the one of humans in recognizing emotions from masked faces, we found that the system outperforms the capability of the human annotators (+ 14%).

Despite the encouraging results, there are still some issues that limit the application of our FER system. The main limitations are related to the fact that, in its current version, the system is able to recognize emotions from frontal face images. Indeed the approach should be tested on more naturalistic emotional facial expressions thus dealing with the recognition of emotions even in non-frontal images [40]. For this purpose, other datasets could be taken into account that capture images in-the-wild [22]. Other limitations concern the use of static images for training the system, that prevent the system to recognize facial expressions in a dynamic changing environment. Moreover, taking into account also contextual sources of information, such as the body position and posture or the scene surrounding the person can be useful for solving disambiguation and improving the performance. As future work, we plan to improve our pipeline by adopting a model that is able to manage video streams. Moreover, we tested our method on masked faces created in an artificial way. In the future, it would be interesting to test the pipeline with real masked faces.

## Data Availability

The data that support the findings of this study are publicly available in the following online repositories and maintained by their respective owners: ∙ https://github.com/prajnasb/observations ∙ https://www.kaggle.com/competitions/challenges-in-representation-learning-facial-expression-recognition-challenge ∙ https://github.com/KDDI-AI-Center/LFW-emotion-dataset

## Footnotes

1 Dlib: http://dlib.net/

2 https://github.com/prajnasb/observations
